# A Metal-Based Inhibitor of NEDD8-Activating Enzyme

**DOI:** 10.1371/journal.pone.0049574

**Published:** 2012-11-19

**Authors:** Hai-Jing Zhong, Hui Yang, Daniel Shiu-Hin Chan, Chung-Hang Leung, Hui-Min Wang, Dik-Lung Ma

**Affiliations:** 1 State Key Laboratory of Quality Research in Chinese Medicine, Institute of Chinese Medical Sciences, University of Macau, Macao, China; 2 Department of Chemistry, Hong Kong Baptist University, Kowloon Tong, Hong Kong, China; 3 Department of Fragrance and Cosmetic Science, Center of Excellence for Environmental Medicine, Kaohsiung Medical University, Kaohsiung, Taiwan; Medical School of Hannover, United States of America

## Abstract

A cyclometallated rhodium(III) complex [Rh(ppy)_2_(dppz)]^+^ (**1**) (where ppy = 2-phenylpyridine and dppz = dipyrido[3,2-*a*:2′,3′-*c*]phenazine dipyridophenazine) has been prepared and identified as an inhibitor of NEDD8-activating enzyme (NAE). The complex inhibited NAE activity in cell-free and cell-based assays, and suppressed the CRL-regulated substrate degradation and NF-κB activation in human cancer cells with potency comparable to known NAE inhibitor MLN4924. Molecular modeling analysis suggested that the overall binding mode of **1** within the binding pocket of the APPBP1/UBA3 heterodimer resembled that for MLN4924. Complex **1** is the first metal complex reported to suppress the NEDDylation pathway via inhibition of the NEDD8-activating enzyme.

## Introduction

The serendipitous discovery of the chemotherapeutic properties of the now well-known anticancer drug cisplatin has aroused considerable interest in the area of medicinal inorganic chemistry [1]–[8]. Cisplatin or its analogues bind DNA and disrupt its double helical conformation, thereby impairing DNA transcription or replication processes and ultimately promoting cell death [9]. However, the adverse side effects and drug resistance associated with the prolonged use of cisplatin has prompted the development of novel bioactive metal complexes displaying distinct mechanisms of action to complement the existing arsenal of platinum-derived cytotoxics.

The application of rhodium complexes as chemotherapeutics has attracted much less attention in contrast to their ruthenium and iridium congeners [10]–[17]. Notable examples of cytotoxic rhodium complexes include the dirhodium(II,II) paddlewheel derivatives [12], [18]–[20] that possess potent *in vitro* activities on a number of cancer cell lines. These complexes display strikingly different coordinative modes to double-helical DNA compared to cisplatin [21], [22], and they have also been reported to interact with proteins [16], [23], presumably through covalent adduct formation with histidine [23], [24] or cysteine residues [23], [25]. Meanwhile, recent research has demonstrated mononuclear rhodium(III) complexes can also be utilized as a molecular scaffold for the construction of structurally complex metal-based enzyme inhibitors that offer comparable potency to organic small molecules [17].

**Figure 1:**
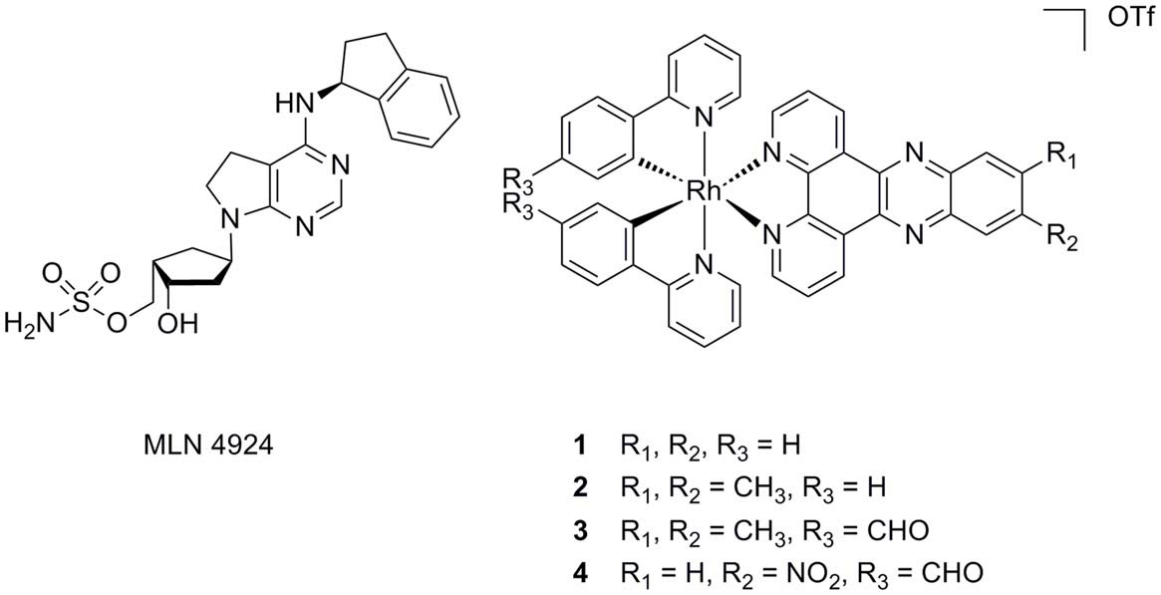
Chemical structures of MLN4924, [Rh(ppy)_2_(dppz)]^+^ (1) and Rh(III) analogues (2–4).

The NEDD8 pathway has recently emerged as a new target for the treatment of cancer [26]–[33]. Modification of the cullin-RING ubiquitin E3 ligases (CRLs) by NEDD8, a ubiquitin-like protein, is known to be essential for the CRL-mediated ubiquitination of downstream targets in the ubiquitin-proteasome system [34], [35], which is critically involved in protein homeostasis. The NEDD8-activating enzyme (NAE) plays an analogous role to the ubiquitin E1 enzyme [36]. NAE is involved in the first step of CRL activation, through activation of NEDD8 and its subsequent transfer to Ubc12, the E2 conjugating enzyme of the NEDD8 pathway. NEDD8 then becomes conjugated to a conserved lysine residue near the C-terminus of the cullin proteins of the CRLs. This covalent modification is required for the cullin complex to recruit an ubiquitin-charged E2 enzyme in order to facilitate the polyubiquitination of proteins, yielding substrates for proteasomal degradation [37]–[42]. Thus, the targeted inhibition of NAE could mediate the rate of ubiquitination and the subsequent degradation of substrates regulated by CRLs, such as IκBα and p27. These proteins have important roles in DNA replication and repair, NF-κB signal transduction, cell cycle regulation and inflammation. Targeting a specific E3 such as the CRLs compared to a more upstream enzyme would have the potential to only stabilize a particular subset of proteins, possibly resulting in an improved selectivity profile [43]. The NAE inhibitor MLN4924 [43] (Figure 1) was recently reported to be effective against both solid (colon, lung) and hematological (myeloma, lymphoma) human cancer cells. We have previously employed high-throughput virtual screening to identify 6,6′′-biapigenin as only the second inhibitor of NEDD8-activating enzyme from a natural product and natural product-like database [44]. While transition metal complexes have been widely utilized for the treatment of cancer [45]–[47], their activity against NEDD8-activating enzyme has not been explored. Inspired by the above findings as well as pioneering works from the Meggers’s group on the design of structurally rigid octahedral ruthenium(II) [48]–[53] and iridium(III) [53]–[55] complexes as shape-complementary inhibitors of protein kinases, we sought to investigate the biological effects of a series of cyclometallated rhodium(III) complexes on the NEDD8 pathway. Cyclometallated rhodium(III) complexes containing the dipyrido[3,2-*a*:2′,3′-*c*]phenazine dipyridophenazine (dppz) scaffold were chosen because of the following reasons: 1) the rhodium(III) complex adopts an octahedral geometry rather than a square planar or tetrahedral symmetry, thus allowing much larger structural complexity for potential use in drug design; 2) the octahedral geometry of the rhodium complex provides a globular and rigid scaffold with limited conformational freedoms of the co-ligands that may interact with the previously inaccessible regions of chemical space in NAE; 3) the synthetic route for **1** is modular and convenient, thus allowing structural modification without the need for lengthy synthetic protocols; and 4) the extended aromatic dppz ligand structurally resembles the planar nature of NAE inhibitor 6,6′′-biapigenin [44], potentially functioning as the recognition arm for NAE.

We report herein the synthesis and characterization of the racemic mixture of rhodium(III) complex [Rh(ppy)_2_(dppz)]^+^ (**1**) and its analogues (**2**–**4**). Complex **1** was found to inhibit NAE activity *in vitro* and *in cellulo*. We then investigated the structure-activity relationship of the Rh(III) complexes against NAE activity *in vitro*. Furthermore, complex **1** inhibited downstream CRL-regulated substrate degradation and NF-κB signaling *in cellulo*, and was also found to exhibit prominent anti-proliferative activity against a human cancer cell line. Molecular modeling analysis revealed that **1** occupied the same binding pocket as MLN4924, the most potent NAE inhibitor to date.

## Results and Discussion

The precursor complexes [Rh(ppy)_2_(OH_2_)_2_]^+^ (ppy = 2-phenylpyridine) and [Rh(fppy)_2_(OH_2_)_2_]^+^ (fppy = 4-(2-pyridyl)benzaldehyde), dppz, and dppz analogues were prepared according to a modified literature method [56]–[58]. Complex **1** was prepared by treating the precursor complex [Rh(ppy)_2_(OH_2_)_2_]^+^ with dppz in refluxing MeCN, and was precipitated as the CF_3_SO_3_^–^ salts. Complexes **2**–**4** were synthesized in a similar fashion using the corresponding precursor complexes and ligands (Figure S1). The structures and the purities of complex **1–4** were determined using NMR spectroscopy and high resolution mass spectrometry.

**Figure 2:**
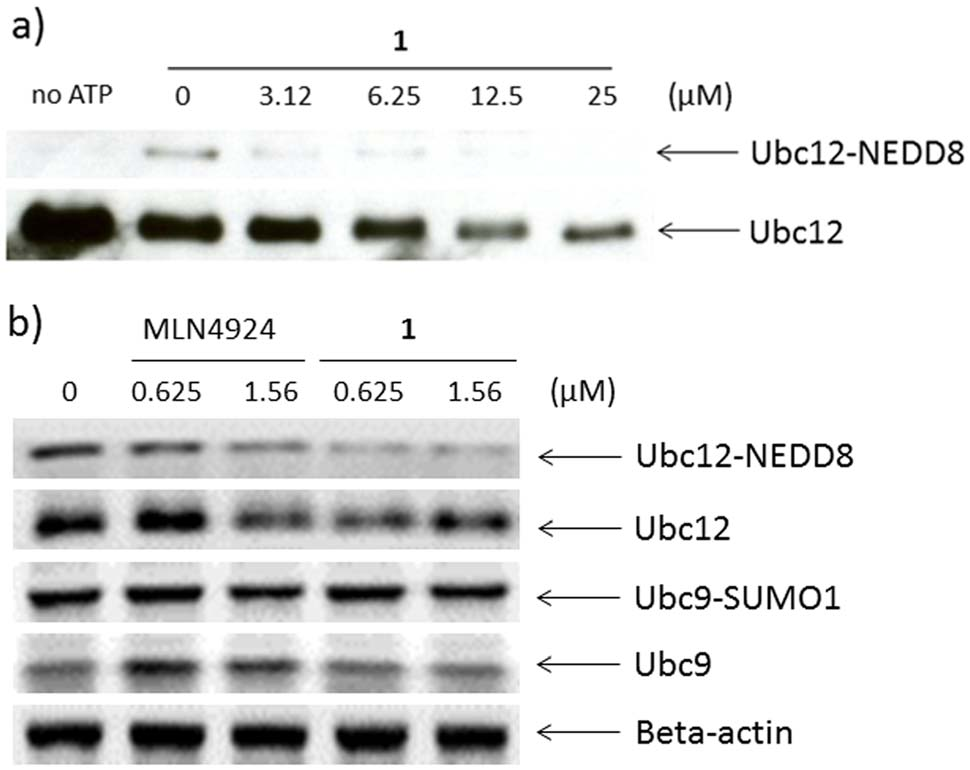
Complex 1 inhibits NAE activity in a dose-dependent manner. Western blots show dose-dependent inhibition of a) Ubc-12-NEDD8 formation in a cell-free system and b) cellular Ubc12-NEDD8 levels by **1**. MLN4924 was included for comparison.

The impact of **1** on the NAE activity was first evaluated by a cell-free assay that measures the formation of the Ubc12-NEDD8 conjugation product. Recombinant human NAE was incubated with Ubc12 and NEDD8 in the presence of vehicle (DMSO) or **1**. NAE promotes NEDDylation of Ubc12 which leads to the formation of the Ubc12-NEDD8 thioester product. We observed that this process was suppressed in the presence of **1**. Western blot analysis revealed a dose-dependent reduction of Ubc12-NEDD8 formation by **1** with an IC_50_ value of *ca.* 1.5 µM and complete inhibition at 12.5 µM (Figure 2a).

We next evaluated the ability of **1** to inhibit Ubc12-NEDD8 formation inside human epithelial colorectal adenocarcinoma (Caco-2) cells. A dose-dependent decrease in the level of the Ubc12-NEDD8 conjugate was observed upon treatment with **1** for 16 hours (Figure 2b). However, no significant inhibition of Ubc9-SUMO formation was observed at the same concentrations tested. The potency of **1** against NAE activity in cells was comparable to that of known inhibitor MLN4924 under the conditions employed. This result shows that complex **1** was able to suppress the activity of NAE in a cellular system but not the closely related SAE, which is consistent with the result from cell-free western blotting.

We then screened three analogues of complex **1** in order to establish a brief structure-activity relationship for the observed *in vitro* effects against NAE activity in the cell-free assay (Figure S2). Interestingly, the results showed that functionalizing the dppz ligand with methyl groups (complex **2**) or the ancillary ppy ligands with aldehyde groups (complex **3**) yielded less active analogues (IC_50_ = *ca.* 6.25 µM). However, the incorporation of an electron-withdrawing nitro group on the dppz motif together with aldehyde functionalities at the ppy ligands (complex **4**) resulted in the weakest NAE inhibitory activity (IC_50_ = *ca.* 13 µM). Furthermore, the uncoordinated dppz ligand was found to be inactive against Ubc12-NEDD8 conjugation at a relative high concentration tested (data not shown). Taken together, these data suggest that the metal center plays an important role in the arrangement of ligands for optimal recognition of the protein binding site, and that the observed *in vitro* potency of these analogues are sensitive to steric and/or electronic properties of the metal complexes.

NAE promotes ubiquitination and the subsequent degradation of a subset of proteins regulated by CRLs such as IκBα [59], [60] and p27 [61], [62]. The IκBα protein plays a central role in repressing the activity of the transcription factor NF-κB [63], [64], which is involved in important cellular processes including the immune response, programmed cell death, as well as cancer initiation and progression [65], [66]. The inhibition of NAE is therefore a potential approach to block the degradation of IκBα thus preventing NF-κB activation. On the other hand, the p27 is a cell cycle regulator which controls cell cycle progression during the G1 phase [67]. The loss of function in p27 can lead to uncontrolled cell proliferation and the development of cancer.

**Figure 3:**
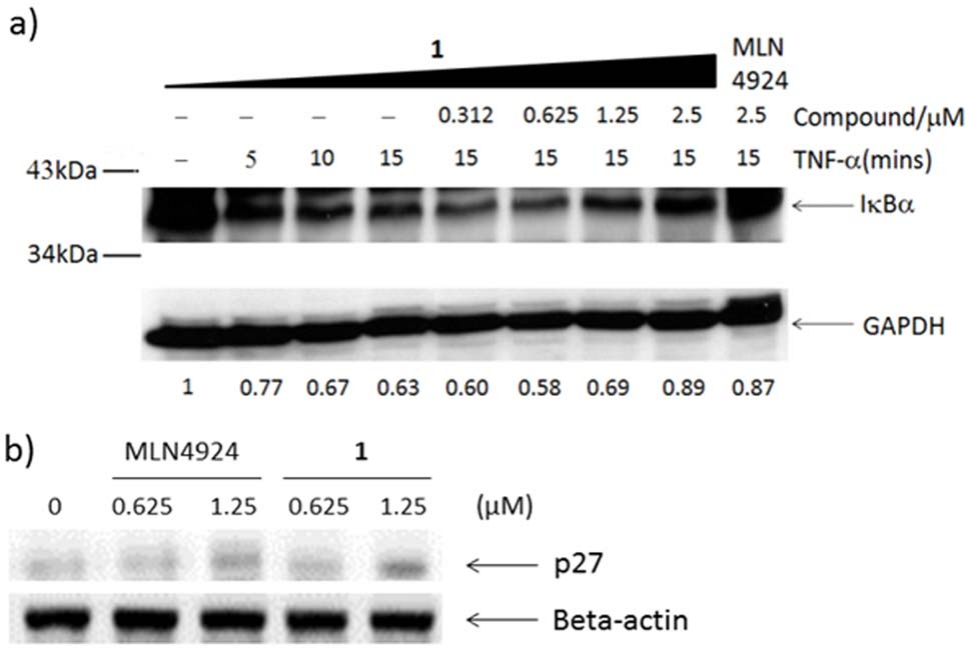
Dose-dependent inhibition of IκBα degradation by 1. Caco-2 cells were pre-incubated with indicated concentrations of **1** for 16 hours and then stimulated with 5 ng/ml of TNF-α at indicated time intervals. Whole cell lysates were analyzed by Western blot using anti-IκBα antibody. Densitometry estimates of IκBα levels normalized with GAPDH are shown under each lane. b) Caco-2 cells were treated with **1** or MLN4924 for 16 h. The cell lysates were immunoblotted to analyse the level of CRL substrate p27.

Inhibition of NAE activity in Caco-2 cells by **1** should be expected to result in the down-regulation of the CRL activity thus leading to the accumulation of CRL substrates [43]. To examine this, we first investigated the ability of **1** to inhibit NAE-regulated IκBα degradation. Caco-2 cells were stimulated with TNF-α to induce IκBα protein degradation, which was monitored using Western blot analysis (Figure 3a). Encouragingly, we observed that the induction of IκBα protein degradation by TNF-α was blocked by **1** in a dose-dependent manner, with potency comparable to the control compound MLN4924 at 2.5 µM. Similarly, treatment of Caco-2 cells with **1** resulted in accumulation of p27 protein, with comparable effects to MLN4924. In summary, complex **1** has been found to block the degradation of CRL substrates (i.e. IκBα and p27), presumably via its ability to inhibit NAE activity.

**Figure 4:**
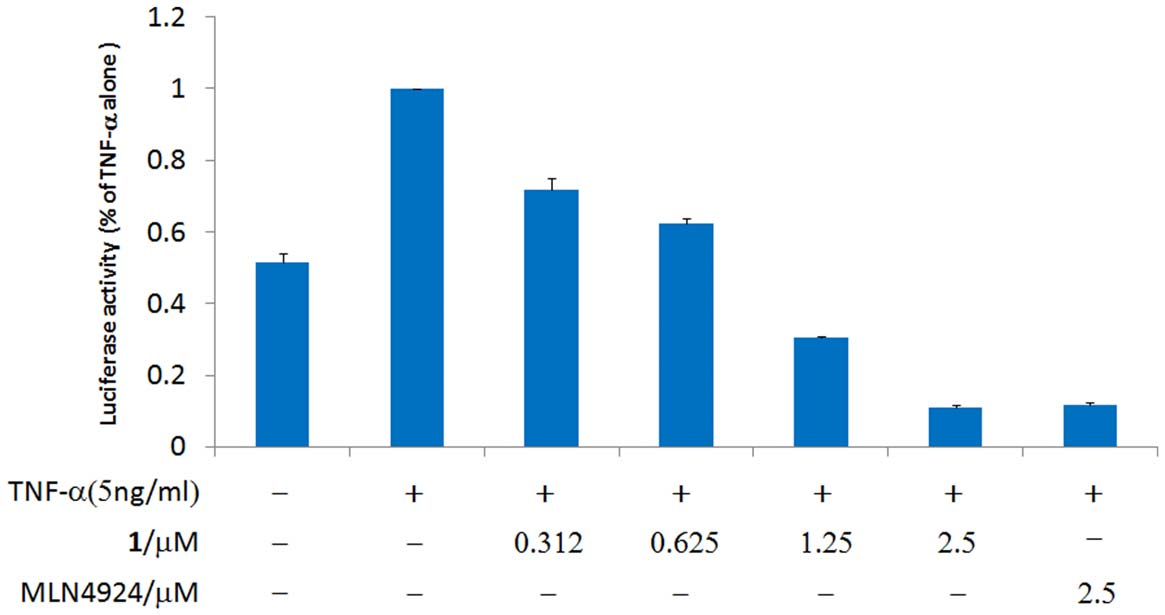
Complex 1 suppresses NAE-regulated NF-κB-dependent luciferase reporter gene expression in a dose-dependent manner. Caco-2 cells were transfected with the NF-κB-dependent luciferase reporter p3EnhConA-Luc gene, treated with **1** for 16 hours, and then stimulated with 5 ng/ml of TNF-α for 3 hours. Luciferase expression was measured and normalized with β-galactosidase activity. Results are expressed as fold change compared to TNF-α stimulation alone and the errors bar show the standard derivation of triplicate results. MLN4924 was included for comparison.

Since the accumulation of IκBα would be expected to repress NF-κB activity, we next investigated the ability of complex **1** to interfere with NF-κB signaling in human cells. Caco-2 cells transfected with the luciferase reporter plasmid harboring NF-κB-luciferase gene were pre-incubated with **1** prior to TNF-α activation. The inhibition of TNF-α-induced NF-κB signaling is manifested as a reduction in luciferase activity. We observed a dose-dependent reduction of NF-κB activity by **1**, with an estimated IC_50_ value of *ca.* 0.77 µM (Figure 4). The potencies of **1** and MLN4924 were found to be similar in a parallel experiment, with 90% inhibition of NF-κB activity at a concentration of 2.5 µM. These results suggest that complex **1** inhibits NF-κB signaling in human cells, and are consistent with the observed accumulation of IκBα induced by **1** as described previously.

The cytotoxicity of complex **1** towards human cancer cells was investigated using an MTT assay. Complex **1** inhibited the growth of the Caco-2 cells with an IC_50_ value of *ca.* 0.3 µM, which was approximately ten-fold more potent than MLN4924 in a parallel experiment (Figure S3). We believe that the cytotoxicity of **1** against cancer cells is due, at least in part, to its ability to up-regulate p27 protein level and attenuate NF-κB signaling as described previously. p27 is a cell cycle inhibitor and the accumulation of this protein should be expected to slow cell division. Furthermore, impaired NF-κB signaling should result in increased susceptibility to cell death due to the role of NF-κB in regulating anti-apoptotic genes. However, further investigation into the exact mechanism of cytotoxicity by complex **1** is required to elucidate the relative contribution of NAE inhibition towards the observed cytotoxic effects of this complex.

**Figure 5:**
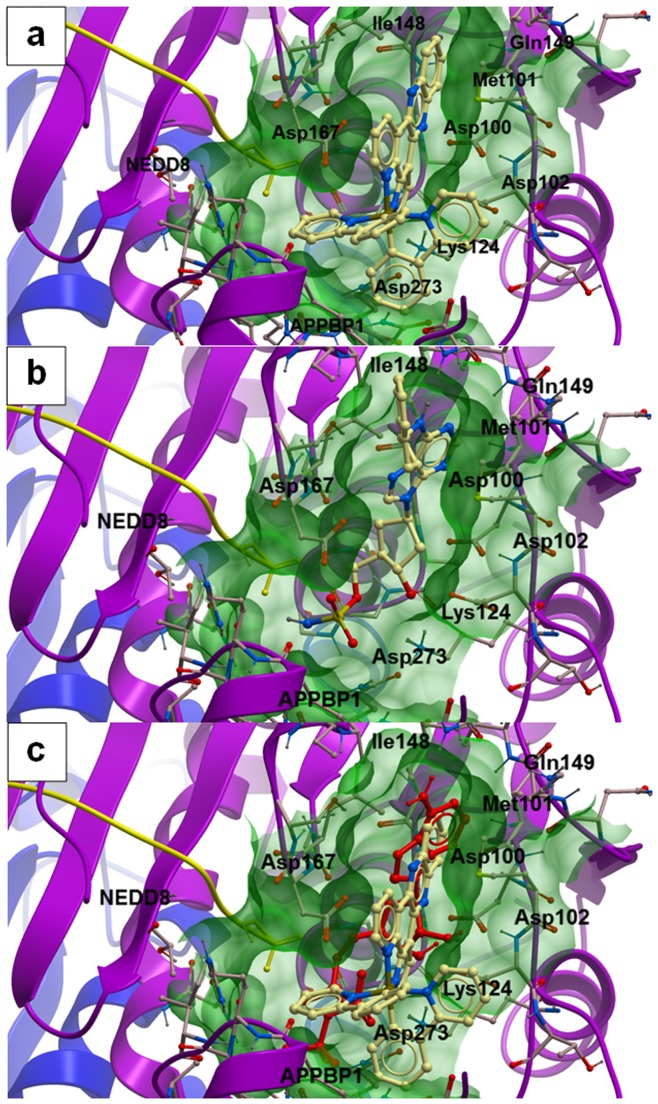
Low-energy binding conformations of a) 1, b) MLN4924 and c) both 1 and ATP bound to NAE heterodimer generated by virtual ligand docking. Proteins APPBP1 (blue), UBA3 (purple) and NEDD8 (yellow) are displayed in ribbon form. Small molecules are depicted as a ball-and-stick model showing showing carbon (yellow), hydrogen (grey), oxygen (red), nitrogen (blue), phosphorus (orange) and sulfur (green). Non-polar hydrogens were not shown.

We performed molecular modeling to investigate the possible binding interactions of **1** in the binding pocket of the NAE-NEDD8 complex. We envisioned that the octahedral geometry of complex **1** may populate previously inaccessible regions of chemical space in the UBA3 and APPBP1 subunits of NAE (Figure 5a). The molecular docking results showed that the highest-scoring binding pose of complex **1** overlapped considerably with that of MLN4924 (Figure 5b) [68]. The dppz ligand of **1** was predicted to occupy the hydrophobic pocket near Met101 and Ile148 as well as the ribose binding region located between Asp100 and Asp 167, contacting similar residues as the indan and dihydropyrrolopyrimidine systems of MLN4924. We presumed that the structural and electronic similarity between the dppz moiety and the aromatic ring systems of MLN4924 possibly contributes to the favourable binding interaction between the rhodium(III) complex and UBA3 subunit. The rhodium(III) metal centre is situated in the central canyon-like groove of NAE in a similar region to that occupied by the γ-phosphate group of ATP. Interestingly, the three-dimensional structural arrangement of the ligands means that one of the phenylpyridine moieties of **1** is closer to the APPBP1 subunit of NAE compared to MLN4924, allowing potential hydrophobic interactions to form with the residues near Lys124 and Asp273. On the other hand, the other phenylpyridine ligand is located closer to NEDD8, in a similar area to that of the ribose ring of MLN4924. The lowest-energy binding mode of **1** was also significantly similar to that for ATP (Figure 5c). For reference, the binding score for **1** with NAE was calculated to be –32.89, compared to –30.3 and –30.8 for ATP and MLN4924, respectively. Based on the strong calculated binding score of **1** to the active site of NAE, as well as the multiple Van der Waals interactions predicted between **1** with the UBA3, APPBP1 and NEDD8 subunits, we propose that **1** may act as a reversible ATP-competitive inhibitor of NAE by occupying the ATP-binding domain. Other possible binding poses of **1** and their corresponding docking scores are also included in the Supporting Information (Table S1).

### Conclusion

In summary, we have identified the rhodium(III) complex **1** as a new inhibitor of NAE. The identification of the metal-based inhibitor **1** represents, to our knowledge, the first reported example of NAE inhibition by a transition metal complex and only the third example of a small-molecule inhibitor of NAE. Complex **1** was found to inhibit NAE activity in a cell-free assay and also reduced Ubc12-NEDD8 conjugate levels in human cancer cells. Significantly, complex **1** blocked CRL substrate degradation and repressed NF-κB activation in human cancer cells with comparable potency to MLN4924, the strongest NAE inhibitor reported to date. Our brief structure-activity relationship analysis and molecular modeling results suggest that the unique structural features of the octahedral coordination geometry of the Rh(III) complex **1** allows it to form optimal interactions with NAE, which is envisaged to contribute significantly to its binding potency and selectivity for NAE over the closely-related enzyme SAE. Based on our findings, we believe that this bioactive complex can potentially be developed as a useful lead to generate more potent analogues for chemotherapeutic or autoimmune/inflammatory applications.

## Materials and Methods

### Material and Cell Lines

All the chemicals, unless specified, were purchased from Sigma-Aldrich and were used as received. NEDD8 Conjugation Initiation Kit and anti-Ubc12 rabbit polyclonal antibody was obtained from Boston BioChem (Cambridge, MA, USA). Caco-2 cells were purchased from American Type Culture Collection (Manassas, VA, USA), catalog number: HTB-37. Cells were cultured in Minimum Essential Media containing 10% fetal bovine serum and were incubated at 37°C/5% CO_2_. Deuterated solvents for NMR purposes were obtained from Armar and used as received.

### General Experimental

All ^1^H and ^13^C NMR spectra were recorded on a Bruker Avance 400 spectrometer operating at 400 MHz. The ^1^H and ^13^C chemical shifts were referenced internally to solvent shift (CDCl_3_: ^1^H δ 7.26, ^13^Cδ 77.2; MeOD: ^1^H δ 3.31, ^13^C δ 49.15; d_6_-DMSO: ^1^H δ 2.50, ^13^C δ 39.5; CD_3_CN:^ 1^H, δ 1.94, ^13^C δ 118.7). Chemical shifts are quoted in ppm, the downfield direction being defined as positive. Uncertainties in chemical shifts are typically ±0.01 ppm for ^1^H and ±0.05 for ^13^C. Coupling constants are typically ±0.1 Hz for ^1^H-^1^H and ±0.5 Hz for ^1^H-^13^C couplings. The following abbreviations are used for convenience in reporting the multiplicity of NMR resonances: s, singlet; d, doublet; t, triplet; q, quartet; m, multiplet; br, broad. All NMR data were acquired and processed using standard Bruker software (Topspin). MALDI-MS analysis was performed using a Bruker Autoflex II mass spectrometer (Bruker Daltonics, Germany) equipped with a nitrogen laser (337 nm, wavelength; 3 ns pulse width) operated in reflectron mode with accelerating voltage, grid voltage and delayed extraction time set to 19 kV, 90%, and 120 ns, respectively. Unless otherwise stated, each mass spectrum was acquired as an average of 200 laser shots at 10.0 Hz frequency.

### Synthesis of Rhodium(III) Complexes

#### Preparation of dipyrido[3,2-*a*:2′,3′-*c*]phenazine phenazine (dppz) derivatives

A mixture of 1,10-phenanthroline-5,6-dione (2 mmol) and appropriate *o*-phenylenediamine derivate (2.4 mmol) in ethanol (200 mL) was stirred at 50°C for 2 h and then at room temperature overnigt. The precipitate was collected by filtration and washed with cold ethanol (3 × 15 mL). The crude product was then recrystallized from methanol.

#### 11-Nitrodipyrido[3,2-*a*:2′,3′-*c*]phenazine

Yellow solid, 557.1 mg, 85.1%. ^1^H NMR (400 MHz, CDCl_3_): 9.57 (t, J = 8.0 Hz, 2H), 9.30 (t, J = 8.0 Hz, 2H), 9.23 (s, 1H), 8.65 (d, J = 8.0 Hz, 1H), 8.46 (d, J = 8.0 Hz, 1H), 7-83-7.80 (m, 2H). ^13^C NMR (400 MHz, CDCl_3_): 153.9, 153.7, 149.3, 148.9, 148.4, 144.4, 143.7, 143.3, 141.1, 134.5, 134.3, 131.4, 127.0, 126.9, 126.2, 124.8, 124.7, 123.9. Maldi-TOF-HRMS: Calcd for C_18_H_9_N_5_O_2_ [M+H]^+^: 328.0829 Found: 328.0813.

#### 11,12-Dimethyldipyrido[3,2-*a*:2′,3′-*c*]phenazine

Light yellow solid, 541.9 mg, 87.3%. ^1^H NMR (400 MHz, CDCl_3_): 9.51 (d, J = 8.0 Hz, 2H), 9.22 (d, J = 4.0 Hz, 2H), 7.94 (s, 2H), 7.75-7.725 (m, 2H), 2.53 (s, 6H). ^13^C NMR (400 MHz, CDCl_3_): 152.3, 148.3, 141.9, 141.7, 140.3, 133.6, 128.3, 127.9, 124.1, 20.8. Maldi-TOF-HRMS: Calcd for C_20_H_14_N_4_ [M+Na]^+^: 333.1111 Found: 333.1097.

#### Preparation of the precursor complexes b and c

A solution of rhodium(III) chloride (200 mg, 0.88 mmol) and 2-phenylpyridine (275.74 µL, 1.93 mmol, 2.2 eq.) in a mixture of methoxyethanol : water (3∶ 1, 48 mL) was heated under reflux overnight under a nitrogen atmosphere. The reaction mixture was cooled to room temperature. The yellow solid was collected by filtration, washed with additional portions of water (2 × 100 mL) and diethyl ether (2 × 50 mL) and dried in vacuo to yield the compound a (230 mg, 58.5%). The suspension of a (100 mg, 0.12 mmol) and silver triflate (166.8 mg, 0.64 mmol, 5.8 eq.) in a mixture of ethanol : water (9∶ 1, 20 mL) was refluxed under a nitrogen atmosphere overnight. The solvent was removed in vacuo and the residue was dissolved in dichloromethane (*ca.* 50 mL) and filtered. The filtrate was removed in vacuo to yield the corresponding precursor complex.

#### Complex b

Brown solid, 100 mg, 74.9%. ^1^H NMR (400 MHz, MeOD): δ 8.83 (d, J = 5.2 Hz, 2H), 8.23-8.14 (m, 4H), 7.88 (d, J = 7.6 Hz, 2H) 7.59-7.55 (m, 2H), 7.07 (t, J = 7.6 Hz, 2H), 6.91 (t, J = 7.2 Hz, 2H), 6.26 (d, J = 7.6 Hz, 2H). ^13^C NMR (400 MHz, MeOD): 165.2, 158.6 (d, J = 38.1 Hz), 150.6, 146.3, 140.7, 132.7, 131.0, 126.2, 125.2, 124.8, 121.3. Maldi-TOF-HRMS: Calcd for C_22_H_16_N_2_Rh [M-2H_2_O-OTf]^+^: 411.2824 Found: 411.3287.

#### Complex c

Yellow solid, 166.8 mg, 86.9%. ^1^H NMR (400 MHz, MeOD): 8.86 (d, J = 4.0 Hz, 2H), 8.18-8.13 (m, 4H), 7.75 (d, J = 8.0 Hz, 2H), 7.56 (dt, J = 8.0 Hz, 4.0 Hz, 2H), 7.03 (d, J = 8.0 Hz, 2H), 6.22 (s, 2H). ^13^C NMR (400 MHz, MeOD): 165.6, 150.6, 146.2, 140.6, 132.6, 125.3, 124.6, 123.4, 121.2, 120.3, 104.4. Maldi-TOF-HRMS: Calcd for C_24_H_20_N_2_O_4_Rh [M-2H_2_O-OTf]^+^: 467.0261 Found: 467.0242.

#### Preparation of the complex 1

The solution of **b** (30.4 mg, 0.06 mmol) and dppz (14.4 mg, 0.06 mmol) was refluxed in acetonitrile (50 mL) overnight under a nitrogen atmosphere. The solvent was removed in vacuo and the residues were washed with diethyl ether (2 × 50 mL) to yield the titled compound as a brown solid (19.5 mg, 58.2%). ^1^H NMR (400 MHz, d6-DMSO): 9.77 (d, J = 8.2 Hz, 2H), 8.51–8.48 (m, 2H), 8.34–8.29 (m, 4H), 8.20-8.15 (m, 4H), 8.03–7.95 (m, 4H), 7.61 (d, J = 5.6 Hz, 2H), 7.17–7.01 (m, 6H), 6.30 (d, J = 7.4 Hz, 2H). ^13^C NMR (400 MHz, d_6_-DMSO): 167.7, 167.3, 164.8, 152.0, 150.1, 148.6, 144.5, 142.6, 140.7, 139.6, 136.1, 133.1, 132.8, 130.7 (d, 20.4 Hz), 130.0, 128.7, 125.6, 124.5, 124.1, 120.8. Maldi-TOF-HRMS: Calcd for C_40_H_26_N_6_Rh [M-OTf]^+^: 693.1269 Found: 693.1251.

#### Preparation of the complexes 2–4

The procedure for preparation of complex **1** was adopted using the corresponding precursor complex and the dppz derivative.

#### Complex 2

Yellow solid, 33.1 mg, 63.4%**.** ^1^H NMR (400 MHz, CD_3_CN): 9.76 (dd, J = 8.0 Hz, 4.0 Hz, 2H), 8.41 (d, J = 4.0 Hz, 2H), 8.18 (s, 2H), 8.10 (d, J = 8.0 Hz, 2H), 7.99–7.96 (m, 2H), 7.92–7.86 (m, 4H), 7.57 (d, J = 4.0 Hz, 2H), 7.17 (t, J = 8.0 Hz, 2H), 7.05 (t, J = 8.0 Hz, 2H), 6.95 (t, J = 8.0 Hz, 2H), 6.41 (d, J = 8.0 Hz, 2H), 2.65 (s, 6H). ^13^C NMR (400 MHz, CD_3_CN): 168.4, 168.1, 166.2, 153.1, 151.0, 148.9, 145.4 (d, J = 20.0 Hz), 143.0, 140.2, 140.0, 136.3, 134.1, 131.6, 129.0, 128.9, 126.2, 125.0, 121.5, 21.2. Maldi-TOF-HRMS: Calcd for C_42_H_30_N_6_Rh [M-OTf]^+^: 721.1582 Found: 721.1552.

#### Complex 3

Light yellow solid, 29.8 mg, 53.5%. ^1^H NMR (400 MHz, d6-DMSO): 9.78 (s, 2H), 9.64 (d, J = 8.0 Hz, 2H), 8.53 (d, J = 8.0 Hz, 2H), 8.34–8.31 (m, 4H), 8.16–8.10 (m, 6H), 7.75–7.70 (m, 4H), 7.25 (t, J = 8.0 Hz, 2H), 6.78 (s, 2H), 2.59 (s, 6H). ^13^C NMR (400 MHz, d_6_-DMSO): 193.1, 166.8, 162.5, 151.3, 150.1, 149.6, 147.5, 143.9, 141.1, 139.6, 138.9, 136.2, 135.3, 131.3, 130.2, 128.1, 127.7, 126.7, 125.5, 125.4, 121.9, 20.2. Maldi-TOF-HRMS: Calcd for C_44_H_30_N_6_O_2_Rh [M-OTf]^+^: 777.1480 Found: 777.1462.

#### Complex 4

Yellow solid, 27.6 mg, 48.8%. ^1^H NMR (400 MHz, CD_3_CN): 9.83 (d, J = 8.0 Hz, 2H), 9.77 (s, 2H), 9.29 (d, J = 4.0 Hz, 1H), 8.77 (dd, J = 8.0 Hz, 4.0 Hz, 1H), 8.64 (d, J = 8.0 Hz, 1H), 8.47–8.44 (m, 2H), 8.27 (d, J = 8,0 Hz, 1H), 8.12 (d, J = 8.0 Hz, 2H), 8.05–8.00 (m, 4H), 7.70–7.68 (m, 4H), 7.12 (t, J = 4.0 Hz, 2H), 6.87 (s, 2H). ^13^C NMR (400 MHz, CD_3_CN): 194.1, 167.8, 167.5, 164.5, 154.5 (d, J = 24.0 Hz), 151.5, 151.2, 150.6, 150.2, 150.0, 145.9, 143.7, 143.3, 142.7, 140.7, 138.2, 137.5 (d, J = 92.0 Hz), 134.1, 133.0, 131.6 (d, J = 20.0 Hz), 129.6 (d, J = 24.0 Hz), 127.2 (d, J = 8.0 Hz), 126.6, 126.4 (d, J = 12.0 Hz), 123.1. Maldi-TOF-HRMS: Calcd for C_42_H_25_N_7_O_4_Rh [M-OTf]^+^: 794.1017 Found: 794.1048.

### Cell-free NAE Activity Assay

NAE activity assay was conducted using a NEDD8 Conjugation Initiation Kit (Boston BioChem) according to the manufacturer’s instructions. In brief, NAE, NEDD8, Ubc12 and the indicated concentrations of **1** were mixed in the reaction buffer and incubated for 10 minutes, which was followed by the addition of Mg-ATP solution to initiate the reaction. After incubating the mixture at room temperature for 60 minutes, the reaction was terminated with EDTA and the mixture was electrophoresed under non-reducing conditions on a 12% SDS-PAGE gel. The Ubc12 levels were determined by western blot analysis.

### Cell-based Activity Assay

Cells were cultured in the Minimum Essential Media containing 20% fetal bovine serum, 100 U/ml penicillin, and 100 µg/ml streptomycin at 37°C in humidified 5% CO_2_ atmosphere. Caco2 cells grown in 6-well cell-culture plates were treated with the indicated concentrations of **1** or, for the control set-up, 0.1% DMSO for 16 hours. Cells were washed three times with ice-cold PBS, lysed in RIPA buffer, and incubated on ice for 30 minutes. After centrifugation at 14,000 rpm for 30 minutes at 4°C, the supernatant was collected, and the protein concentration was determined with Bio-Rad protein assay dye reagent (Bio-Rad). Equal protein amounts were separated under non-reducing conditions on a 12% SDS-PAGE gel electrophoresis and subjected to western blot analysis.

To study the effect of 1 on NAE-regulated IκBα degradation, Caco2 cells were pre-treated with 0.1% DMSO (control) or the indicated concentrations of **1** for 16 hours before stimulated with 5 ng/ml of TNF-α at indicated time intervals. Whole cell lysates were harvested as described above, and equal protein samples were fractionated by 12% SDS-PAGE gel electrophoresis and then analyzed by Western blot using anti- IκBα antibody (Santa Cruz).

### Wetern-blot Analysis

Protein samples were transferred to PVDF membranes (GE Healthcare) which were subsequently blocked with 5% milk in TBS with 0.05% tween-20 (TBST). The membranes were washed with TBST and immunoblotted with primary antibodies followed by horseradish peroxidase-conjugated secondary antibodies. Labeled protein spots were visualized by ECL (Amersham Biosciences) according to manufacturer’s guildlines.

### Luciferase Reporter Assay

Caco2 cells were cultured to 80% confluent in a 24-well plate and transiently transfected with p3EnhConA-Luc (0.8 µg) using Lipofectamine 2000 (Invitrogen), co-transfected with β-galactosidase control vector (0.2 µg) as an internal transfection efficiency standard. Transfected cells were pre-incubated with the indicated concentrations of **1** for 16 hours before stimulated by 5 ng/ml of TNF-α for an additional 3 hours. Cells were harvested in GLO lysis buffer (Promega). Relative luciferase activity was measured with a Bright-GLO luciferase assay system and normalized with β-galactosidase activity as measured by Beta-GLO luciferase assay system according to the manufacturer’s instruction (Promega).

### Cytotoxicity Test (MTT (3-(4,5-dimethylthiazol-2-yl)-2,5-tetrazolium Bromide) Assay)

Caco2 cell suspensions were seeded at 4000 cells per well in a 96-well culture microplate and incubated overnight at 37°C. Serial dilutions of **1** were added to each well and the microplate was incubated at 37°C, 5% CO_2_, 95% air in a humidified incubator for 72 hours. After the addition of 10 µL MTT reagent (5 mg/mL) to each well, the microplate was re-incubated at 37°C in 5% CO_2_ for 4 hours. The medium was exchanged with DMSO and incubated at room temperature for 5 minutes with shaking. The absorbance at 570 nm was measured using a microplate reader. The IC_50_ value of **1** was determined by the dose-dependence of the surviving cells after exposure to **1** for 72 hours.

### Molecular Modeling

Molecular docking was performed using the ICM-Pro 3.6-1d program (Molsoft) [69]. According to the ICM method, the molecular system was described with internal coordinates as variables. Energy calculations were based on the ECEPP/3 force field with a distance-dependent dielectric constant. The biased probability Monte Carlo (BPMC) minimization procedure was applied to obtain the global-energy optimization. The BPMC global-energy optimization method consists of: 1) a change in conformation, as a result of the random changes in the free variables according to a predefined continuous probability distribution; 2) the local-energy minimization of analytical differentiable terms; 3) calculation of the complete energy, where the nondifferentiable terms, such as entropy and solvation energy, are included; 4) the acceptance or the rejection of the total energy based on the Metropolis criterion and return to step (1). The binding between the complex **1** and NAE-NEDD8 was evaluated with the use of binding energy, which includes grid energy, continuum electrostatic, and entropy terms. The initial model of NAE was built from the X-ray crystal structure of the quaternary APPBP1-UBA3- NEDD8-ATP complex (PDB: 1R4N) [70], according to a previously reported procedure. [44] Hydrogen and missing heavy atoms were added to the receptor structure followed by local minimization by using the conjugate gradient algorithm and analytical derivatives in the internal coordinates. In the docking analysis, the binding site was assigned across the entire structure of the protein complex. The complex **1** was assigned the MMFF force field atom types and charges, and the generated structure was then subjected to Cartesian minimization. The ICM docking was performed to find the most favorable orientation. The resulting trajectories of the complex between the complex **1** and the quaternary protein complex were energy minimized, and the interaction energies were computed.

## Supporting Information

Figure S1 **Synthetic scheme for the preparation of complexes 1–4.**
(TIF)

Figure S2 **Inhibition of Ubc12-NEDD8 conjugation** ***in vitro*** **by the cyclometallated Rh(III) complexes 2–4.**
(PNG)

Figure S3 **MTT cytotoxicity assay showing the cell viability as a function of the concentration of complex 1 and MLN4924.**
(PNG)

Table S1 **Lower-ranking binding conformations of 1 to NAE and their corresponding docking scores generated by virtual ligand docking.**
(DOCX)
