# Supplementary figures and images for: A Metal-Based Inhibitor of NEDD8-Activating Enzyme
Source: PLoS One. 2012 Nov 19;7(11):e49574. doi: 10.1371/journal.pone.0049574 (PMC3501507; doi:10.1371/journal.pone.0049574)

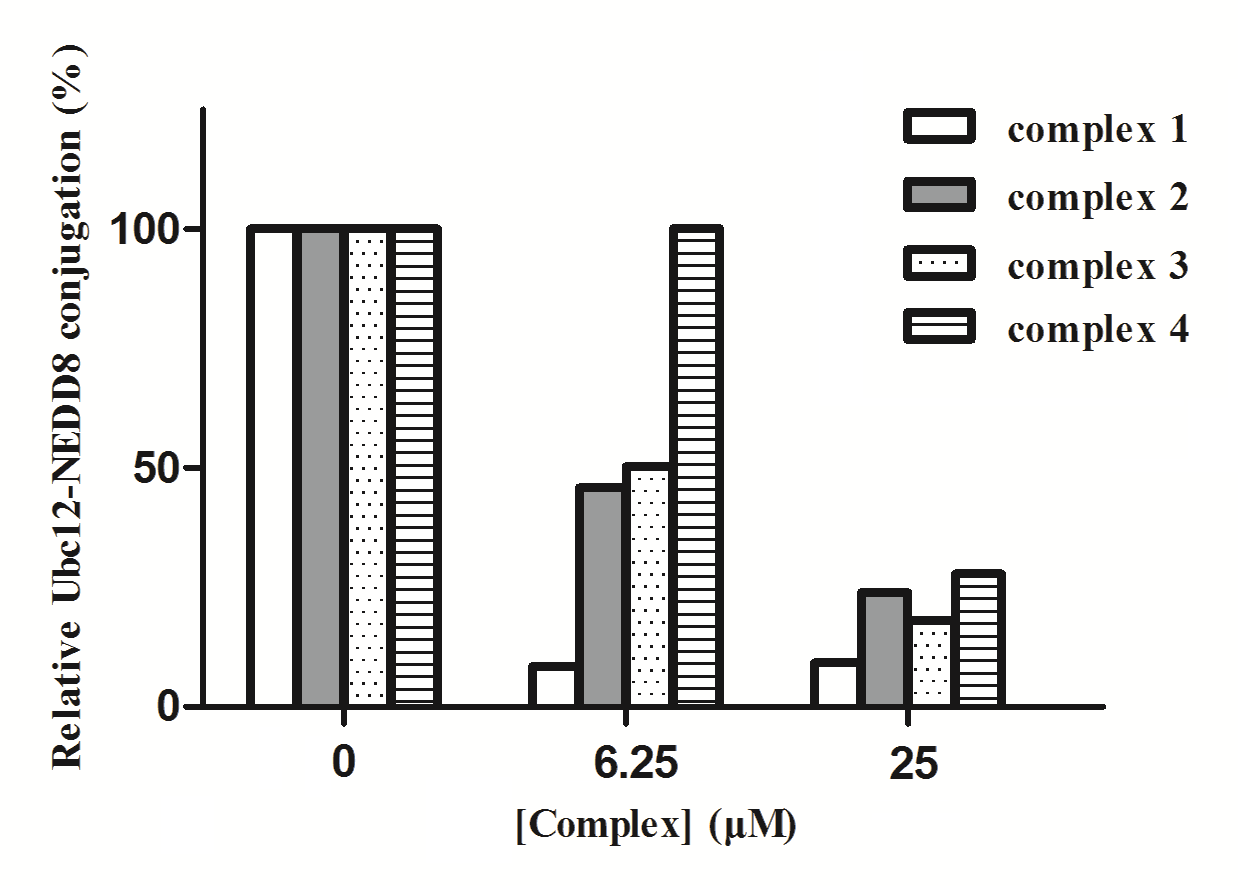

Supplement: Figure S2 — Inhibition of Ubc12-NEDD8 conjugation in vitro by the cyclometallated Rh(III) complexes 2–4. (PNG) [file pone.0049574.s002.png]

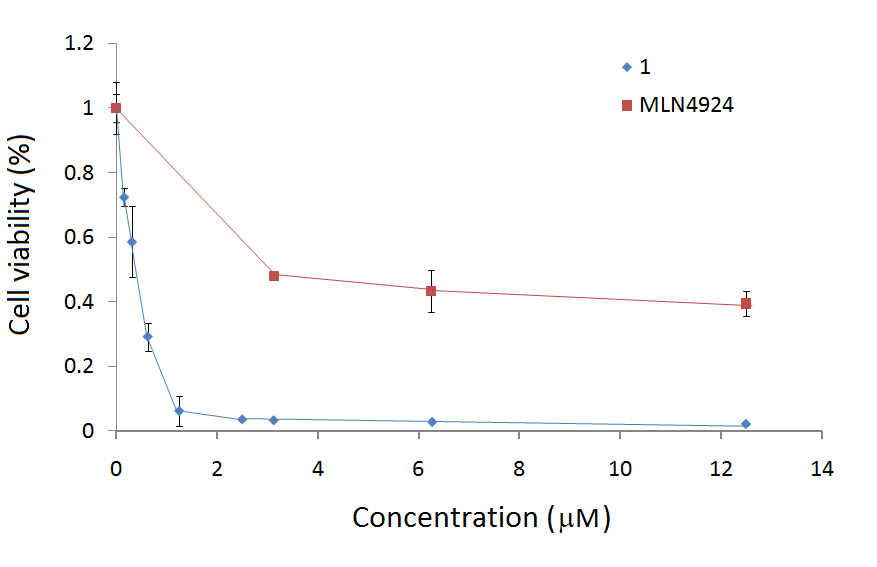

Supplement: Figure S3 — MTT cytotoxicity assay showing the cell viability as a function of the concentration of complex 1 and MLN4924. (PNG) [file pone.0049574.s003.png]
