# Supplementary material for: A Metal-Based Inhibitor of NEDD8-Activating Enzyme
Source: PLoS One. 2012 Nov 19;7(11):e49574. doi: 10.1371/journal.pone.0049574 (PMC3501507; doi:10.1371/journal.pone.0049574)
Supplement: Table S1 — Lower-ranking binding conformations of 1 to NAE and their corresponding docking scores generated by virtual ligand docking. (DOCX) [file pone.0049574.s004.docx]

**Table S1.** Lower-ranking binding conformations of **1** to NAE and their corresponding docking scores generated by virtual ligand docking.

| **Conformation** | **ICM docking score** |
| --- | --- |
| **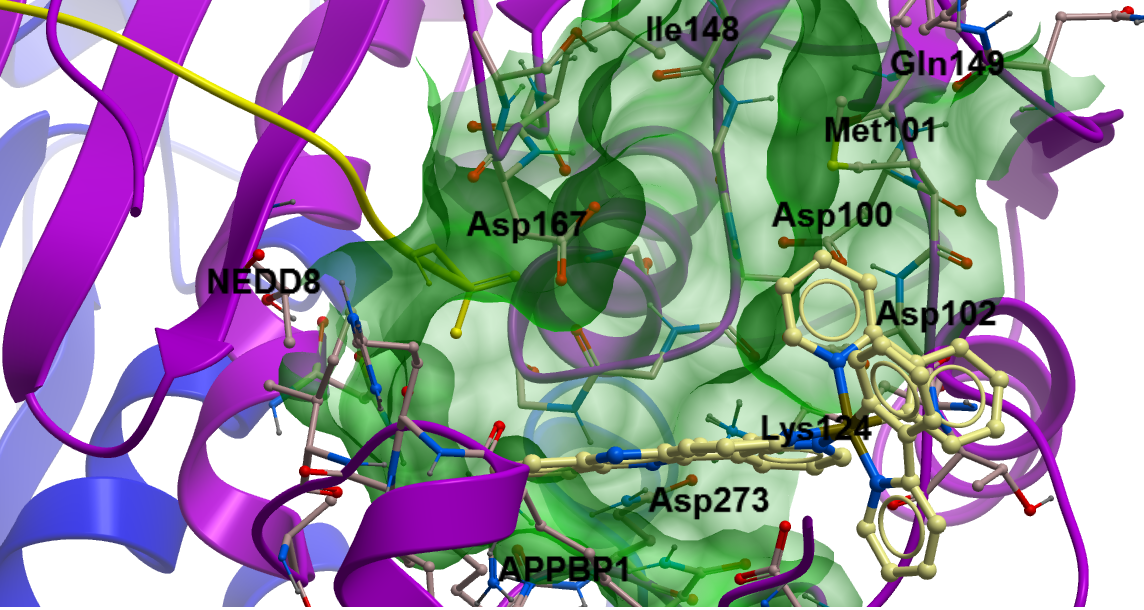** | –28.35 |
| **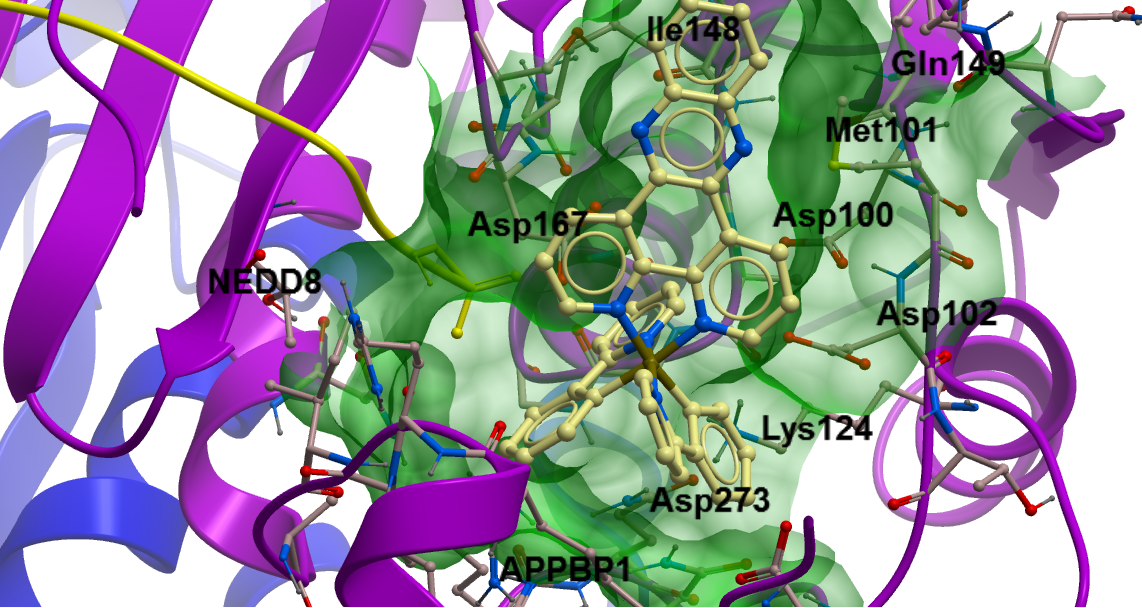** | –25.67 |
| **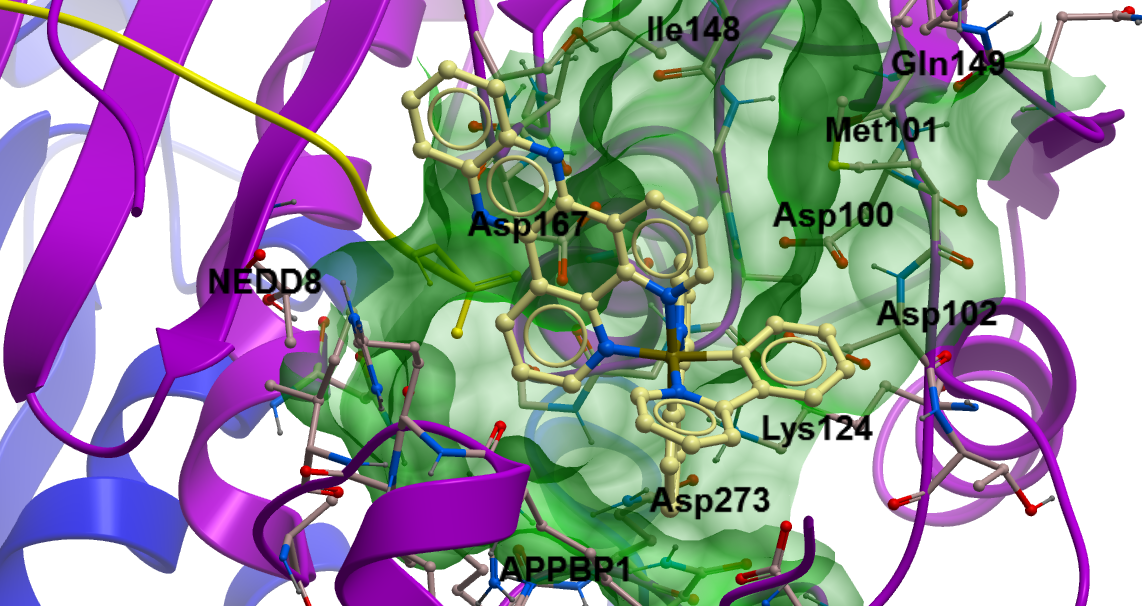** | –24.46 |
